# Mechanisms underlying the emergence of Up and Down states in a model PFC microcircuit

**DOI:** 10.1186/1471-2202-12-S1-O7

**Published:** 2011-07-18

**Authors:** Daphne Krioneriti, Athanasia Papoutsi, Panayiota Poirazi

**Affiliations:** 1 Computational Biology Lab (CBL), Institute of Molecular Biology and Biotechnology (IMBB), Foundation for Research and Technology-Hellas (FORTH), Heraklion, Crete, GR 711 10, Greece; 2 Faculty of Medicine, University of Crete, Heraklion, Crete, GR 710 03, Greece; 3 Biology Department, University of Crete, Heraklion, Crete, GR 714 09, Greece

## 

Up and Down states are oscillations between periods of prolonged activity (Up state) and quiescence (Down state) and are recorded both *in vivo* and *in vitro* in layer V prefrontal cortex (PFC) pyramidal neurons. Biophysical mechanisms that have been proposed to underlie this phenomenon include the balance of excitation and inhibition within local PFC networks [1] along with certain intrinsic membrane mechanisms such as the afterdepolarization [2]. Using a biophysical compartmental network model of PFC layer V pyramidal neurons that incorporates anatomical data (as described in [3]), we investigated the role of synaptic input, intrinsic currents and local interconnectivity in the following features of Up and Down states: (a) the emergence of Up and Down states, (b) the duration of Up states, (c) the frequency of Up states and (d) the firing frequency during the Up state.

We found that Up and Down states could emerge in our model microcircuit (see Figure 1), provided the existence of background synaptic activity. Among the various conditions we examined, statistically significant results were obtained when:

**Figure 1:**
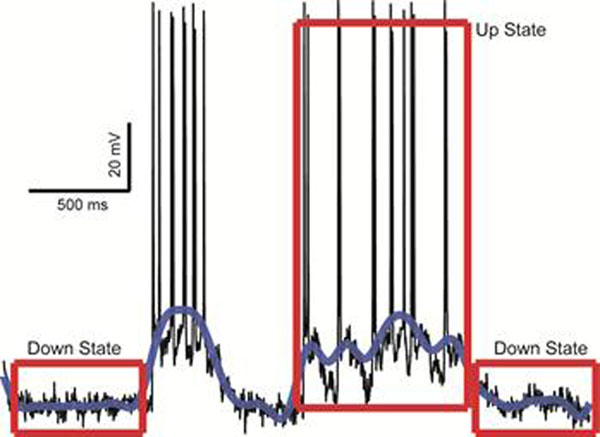
Representative trace (black) of Up and Down states. Blue trace is the signal after it has been filtered with the Butterworth filter. Red boxes are indicative of Down States and an Up state that meets the criteria (500 ms duration and above -60 mV depolarization plateau)

- Increasing the firing frequency of the background synaptic input or the number of activated background synapses (Up frequency increased by ~150% and 60%, respectively, firing frequency increased by ~30% and 50%, respectively).

- Blocking the NMDA current, while compensating for the reduced excitability by enhancing the AMPA current (no emergence of Up and Down states).

- Increasing the iNMDA-to-iAMPA ratio from 1 to 1.5 (Up frequency increased ~190%, firing frequency increased by 25%, Up duration doubled).

- Activating the dADP mechanism at a physiological value (4mV) (Up frequency increased by ~ 200%, firing frequency increased by 60%, Up duration doubled).

## Conclusions

Our results indicate that the generation of Up states in PFC is likely to involve not only a balance of excitation/ inhibition provided within a microcircuit but also single-neuron dynamics shaped by intrinsic mechanisms. Interestingly, the duration of the Up state was significantly altered in only two of the conditions tested, namely, the enhancement of the NMDA current and the activation of the dADP mechanism. These findings suggest that the transition to more prolonged depolarizations is carefully controlled by the same mechanisms that have been associated with persistent firing during working memory tasks.
